# Electrophysiological Subtypes and Prognostic Factors of Guillain-Barre Syndrome in Northern China

**DOI:** 10.3389/fneur.2019.00714

**Published:** 2019-07-02

**Authors:** Jing Tian, Cuifang Cao, Tong Li, Kun Zhang, Peifang Li, Yaling Liu, Xiaoyun Liu

**Affiliations:** Department of Neurology, The Second Hospital of Hebei Medical University, Shijiazhuang, China

**Keywords:** Guillain-Barré syndrome (GBS), acute motor axonal neuropathy (AMAN), acute inflammatory demyelinating polyneuropathy (AIDP), compound muscle action potential with distal stimulation (dCMAP), conduction blocks (CBs)

## Abstract

**Objectives:** To assess the epidemiology of different electrophysiological subtypes of Guillain-Barre syndrome (GBS) and investigate the factors affecting the prognosis of the acute motor axonal neuropathy (AMAN) subtype in northern China.

**Methods:** According to the National Institute of Neurological Disorders and Stroke diagnostic criteria for GBS, 104 consecutive GBS patients were recruited from the Department of Neurology of the Second Hospital of Hebei Medical University, China from 2014 to 2018.

**Results:** Based on nerve conduction studies (NCSs), AMAN was the most common subtype in Northern China, accounting for 58 patients (55.8%). AMAN patients had significantly higher prevalence of antecedent diarrhea, longer duration of hospitalization, and slightly slower recovery than those with acute inflammatory demyelinating polyneuropathy (AIDP), but there was no statistical difference in disease severity or short-term prognosis between AMAN and AIDP. Based on multivariate regression analysis, AMAN patients with antecedent diarrhea (OR = 0.16, 95% CI: 0.03–0.756, *p* = 0.021) or conduction blocks (CBs) (OR = 0.033, 95% CI: 0.001–0.787, *p* = 0.035) had dramatically better short-term prognosis. Decreased compound action potential with distal stimulation (dCMAP) amplitude was associated with significant slower speed of recovery(OR = 8.31, 95% CI: 2.55–27.10, *p* = 0.02).

**Conclusion:** AMAN is still the most common subtype of GBS in northern China. A decline in dCMAP amplitude is predictive factor of a slow recovery and poor outcome of GBS. Diarrhea and CBs may be the factors for better short-term prognosis in AMAN patients in Northern China.

## Introduction

Guillain-Barré syndrome (GBS) is the most common and severe acute paralytic neuropathy, characterized by progressive weakness and diminished or absent deep tendon reflexes. According to neuroelectrophysiological studies, GBS consists of two major subtypes, acute inflammatory demyelinating polyneuropathy (AIDP), and acute motor axonal neuropathy (AMAN). In Europe and the United States, GBS is dominated by AIDP, but in the north of China, especially in Hebei Province, AMAN is thought to be the most common subtype (1). The severity of AIDP and AMAN in different regions has been reported differently. In addition, there are many studies on the factors associated to GBS outcomes (2–4), but these studies include all electrophysiological types, with most patients belonging to the AIDP subtype. Meanwhile AMAN patients present two different patterns of clinical recovery, rapid or slow (5), but the prognosis and related factors for the AMAN subtype is still insufficient. Therefore, this study aimed to investigate the clinical characteristics and the response to intravenous immunoglobulin (IVIg) of the AIDP and AMAN subtypes and explore the prognostic factors in AMAN subtype in Northern China.

## Methods

### Participants

One hundred forty-six participants were selected according to the National Institute of Neurological Disorders and Stroke diagnostic criteria for GBS (6). These patients had NCSs recordings and received sequential treatment during hospitalization in the Department of Neurology of the Second Hospital of Hebei Medical University from January 2014 to April 2018. For further assessment of disease severity and prognostic factors, we excluded patients who were younger than 14 years of age; who had a history of previous GBS or other disease that influenced muscle strength, severe allergic reaction to properly matched blood products, or known selective IgA deficiency; were pregnant; or were receiving immunotherapy outside the hospital. Finally, 104 patients were assessed for disease severity and prognosis-related factors. Clinical data of the 104 patients, including age, sex, season of disease occurrence, antecedent infections (diarrhea or symptoms of upper respiratory tract infection within the 4 weeks preceding the onset of weakness), past medical history (hypertension, diabetes), smoking and drinking status (current and sustained consumption for ≥30 years), tendon reflex, cranial nerve dysfunction, sensory deficits, days between weakness onset and nadir/treatment, nerve conduction results, Hughes Functional Grading Scale (HFGS) score at admission, requirement for mechanical ventilation, treatment modality, HFGS score on day 7 after the first day of immunotherapy, duration of hospitalization, and HFGS score at discharge were analyzed retrospectively. The study protocol was approved by the human ethics committee of the Second Hospital of Hebei Medical University (2018-R250), PR China. Informed consent was obtained from all participants.

### Electrophysiology

An electrophysiological study was performed within 4 weeks of symptom onset. Motor conduction studies were performed to measure the amplitude and duration of the negative peak of the compound muscle action potential (CMAP) with distal (dCMAP) and proximal (pCMAP) stimulation, conduction velocity (CV), distal motor latency (DML), and minimal F-wave latency on the median, ulnar, peroneal, and tibial nerves. The data were finally converted to a percentage of the upper or lower limit of the normal range. For each patient, the normalized data of similar parameters were added together and then divided by the number of data points to obtain the mean of each parameter (7). According to the electrophysiological classification criteria (8), participants were classified into normal subtype, equivocal subtype, AIDP subtype, or AMAN subtype.

### Clinical Score and Evaluation of Short-Term Prognosis

The clinical scores of the 104 patients were evaluated at three time points (on admission, day 7 after the first day of immunotherapy and at discharge) by Hughes Functional Grading Scale (HFGS) score. The HFGS score is used to evaluate the functional status of patient with GBS, it ranges from 0 (normal) to 6 (death) (9). The patients who were unable to walk unaided 5 m across an open space (HFGS score ≥ 3) when discharged (10) were considered as have poor short-term prognosis. Meanwhile, we considered an improvement in HFGS score after 7 days of starting immunotherapy as rapid recovery. Age, dCMAP, days between onset to treatment, dCMAP/pCMAP, and HFGS score at admission and discharge were categorized to facilitate their applicability in clinical practice.

### Treatment

Patients were all treated with IVIg at a dose of 0.4 g/kg/day for 5 days. 39.4% patients received supplementary glucocorticoid therapy. The doses of steroids received by the patients were different: 27 patients (26%) received 5 or 10 mg of dexamethasone for 5 days as anti-allergic treatment before IVIg, while 14 (13.5%) received 500 or 1,000 mg methylprednisolone for 3 days. Meanwhile, after the first round of IVIg, three patients (two AIDP patients and one AMAN patient) had a second round of IVIg treatment within 1 month because of worsening or lack of improvement. All patients were provided appropriate neuro-rehabilitative care and were monitored for complications. Mechanical ventilation was given to patients who did not receive sufficient oxygenation through a nasal cannula or an oxygen storage mask.

### Statistical Analysis

Statistical analysis was performed using SPSS version 25.0 software. Categorical data were presented as proportions, while continuous data were presented as means and standard deviations or means and interquartile ranges depending on their distribution. Differences in proportions were tested by the χ^2^ test. The analysis of numerical variables was performed using the Kruskal-Wallis H test. Potential prognostic factors for outcomes of different electrophysiological subtypes were first analyzed by univariate logistic regression analysis, then statistically significant indicators in the different subtypes were further analyzed in a multivariate logistic model. *P* < 0.05 was considered to be significant.

## Results

### Electrophysiological Categories

A total of 104 patients with NCSs records were included in the electrophysiological studies. Based on electrophysiological criteria, 58 patients (55.8%) were classified as AMAN, 22 (21.2%) as AIDP, 14 (13.5%) as equivocal, and 10 (9.6%) as normal. Of the 104 GBS patients, the clinical characteristics of AIDP (22) and AMAN (58) are compared, as is shown in Table 1. Antecedent diarrhea was statistically significantly different between AIDP and AMAN (*p* = 0.023), and the majority of patients with antecedent diarrhea showed electrophysiological profiles typically belong to AMAN subtype (91.3%). Patients with AIDP were significantly more likely to have cranial nerve involvement (facial paralysis, *p* = 0.06; eyelid ptosis, *p* = 0.02; limited eye muscle activity, *p* = 0.07), and AIDP patients had a higher proportion of smokers (*p* = 0.01). Regarding the NCSs results, there was no statistical difference in dCMAP amplitude between AIDP and AMAN, while the DML of AIDP was significantly longer (*p* < 0.001), the motor nerve conduction velocity of AIDP was dramatically slower (*p* < 0.001). The patients with the AMAN subtype had significantly longer hospital stays than those with AIDP (*p* = 0.01), however, there were no significant differences between the AIDP and AMAN subtypes in mechanical ventilation at admission (*p* = 0.42) or discharge (*p* = 1.00), HFGS score at admission (*p* = 0.94) or discharge (*p* = 0.54), second cycle of IVIg treatment (*p* = 0.18), or combination with intravenous steroids treatment (*p* = 0.59).

**Table 1:** Partial clinical and electrophysiological characteristics of AIDP and AMAN subtype.

**Variable**	**AMAN**	**AIDP**	***P*-value**
Age (years)	52 ± 14.0	54 ± 17.0	NS (0.34)
Sex (male:female)	30:28	16:6	NS (0.09)
Antecedent diarrhea	21 (36.2%)	2 (9.1%)	0.02
Upper respiratory tract infection	22 (37.9%)	10 (45.5%)	NS (0.54)
Facial paralysis	1 (1.7%)	3 (13.6%)	NS (0.06)
Eyelid ptosis	0	3 (13.6%)	0.02
Limited eye muscle activity	0	2 (9.1%)	NS (0.07)
Mechanical ventilation required at admission	3 (5.2%)	3 (13.6%)	NS (0.42)
Mechanical ventilation required at discharge	2 (3.4%)	1 (4.5%)	NS (1.00)
Sensory deficits	18 (31.0%)	12 (54.5%)	0.05
Smoker	1 (1.7%)	5 (22.7%)	0.01
Electrophysiological recordings			
DML	0.89 (0.17)	1.53 (0.72)	<0.0001
dCMAP	0.67 (0.65)	0.53 (1.63)	NS (0.25)
pCMAP	0.40 (0.46)	0.30 (0.36)	NS (0.06)
MNV	1.05 ± 0.10	0.84 ± 0.20	<0.0001
pCMAP/dCMAP	0.65 (0.26)	0.63 (0.32)	NS (0.70)
Second cycle of IVIg treatment	1 (1.7%)	2 (9.1%)	NS (0.18)
Combination of steroids	9 (40.9%)	20 (34.5%)	NS (0.59)
HFGS score ≥3 at admission	48 (82.8%)	18 (81.8%)	NS (0.92)
Improvement after 7 days of treatment	24 (41.4%)	14 (63.6%)	NS (0.08)
Duration of hospitalization	14 (8)	11 (4)	0.01
HFGS score ≥3 at discharge	28 (48.3%)	11 (50.0%)	NS (0.89)

*AMAN, acute motor axonal neuropathy; AIDP, acute inflammatory demyelinating polyneuropathy; DML, distal motor latency; dCMAP, compound muscle action potential with distal stimulation; pCMAP, compound muscle action potential with proximal stimulation; MCV, motor nerve conduction velocity; HFGS, Hughes Functional Grading Scale. Categorical data are presented as proportions, while continuous data are presented as means and standard deviations or means and interquartile ranges depending on their distribution; NS, not significant*.

### Clinical Prognosis

In order to assess the parameters associated with poor outcome, an HFGS score ≥ 3 (suggesting inability to walk unaided across an open space) at discharge or no improvement in the HFGS score 7 days after the first day of immunotherapy were defined as poor outcome. The results by logistic regression analysis were shown in Table 2. For all of electrophysiological subtypes, we found lower dCMAP <0.4 (OR = 8.67, 95% CI: 2.33–32.27, *p* = 0.001), days between onset and treatment >9 (OR = 4.34, 95% CI:1.28–14.66, *p* = 0.018), and HFGS score at admission >3 (OR = 6.88,95% CI:1.49–31.79, *p* = 0.013) were independently associated with poor short-term prognosis in GBS patients. Furthermore, we evaluated the prognostic factors for the AMAN subtype patients alone. Antecedent diarrhea (OR = 0.16, 95% CI: 0.03–0.756, *p* = 0.021) and pCMAP/dCMAP amplitude <0.4 (OR = 0.033, 95% CI: 0.001–0.787, *p* = 0.035) were significantly associated to better prognosis in AMAN patients (Table 3). In addition, a decline in dCMAP amplitude predicted slower recovery in both AIDP and AMAN subtypes (Tables 4, 5).

**Table 2:** Univariate and multivariate regression analyses of the risk of poor short-term prognosis of GBS patients.

**Demographic features**	**Number of patients**	**Patients with poor short-term outcome**	**Univariate OR (95% CI)**	***p*-value**	**Multivariate OR (95% CI)**	***p*-value**
Total	104	42 (40.4%)				
Age, years				0.057		
≤ 50	36	9 (25%)	1			
51–60	36	16 (44.4%)	2.4 (0.88–6.53)			
>60	32	17 (53.1%)	3.4 (1.22–9.48)			
Days between onset to treatment(days)				0.046		0.059
0–4	44	14 (31.8%)	1		1	
5–9	36	13 (36.1%)	1.2 (0.48–3.07)		1.44 (0.47–4.42)	
>9	24	15 (62.5%)	3.57 (1.26–10.12)		4.34 (1.28–14.66)	0.018
pCMAP				0.008		
>1	25	3 (12.0%)	1			
0.40–1	32	14 (43.8%)	5.70 (1.42–22.99)			
0–0.40	47	25 (53.2%)	8.33 (2.19–31.68)			
dCMAP			0.16 (0.07–0.40)	<0.001		0.006
>1	40	8 (20.0%)	1		1	
0.40–1	39	15 (38.5%)	2.50 (0.91–6.85)		2.43 (0.80–7.35)	
0–0.40	25	19 (76.0%)	12.67 (3.81–42.10)		8.67 (2.33–32.27)	
HFGS scores at admission				0.01		0.047
0, 1 or 2	26	3 (11.5%)	1		1	
3	45	18 (40.0%)	5.11 (1.33–19.58)		3.62 (0.84–15.63)	
4 or 5	33	21 (63.3%)	13.42 (3.3–54.22)		6.88 (1.49–31.79)	0.013

*dCMAP, compound muscle action potential with distal stimulation; HFGS, Hughes Functional Grading Scale. Poor short-term prognosis defined as inability to walk unaided (HFGS score ≥3) at discharge*.

**Table 3:** Univariate and multivariate regression analyses of the risk of poor short-term outcome in the AMAN subtype patients.

**Demographic features**	**Number of patients**	**Patients with poor short-term outcome**	**Univariate OR (95% CI)**	***p*-value**	**Multivariate OR (95% CI)**	***p*-value**
Total	58	28 (48.3%)				
No antecedent diarrhea	21	6 (28.6%)	3.67 (1.16–11.60)	0.027	6.29 (1.32–29.87)	0.021
dCMAP				0.037		
>1	15	4 (26.7%)	1			
0.40–1	25	11 (44.0%)	2.16 (0.54–8.68)			
0–0.40	18	13 (72.2%)	7.15 (1.53–33.37)			
pCMAP/dCMAP				0.05		0.11
0–0.40	7	1 (14.3%)	1		1	
0.40–0.80	41	19 (46.3%)	5.18 (0.57–46.96)		6.89 (0.55–86.46)	
0.80–1.00	10	8 (80.0%)	24.00 (1.74–330.80)	0.018	30.20 (1.27–717.60)	0.035
HFGS score at admission				0.118		
0, 1 or 2	10	2 (20.0%)	1			
3	27	13 (48.1%)	3.71 (0.66–20.82)			
4 or 5	21	13 (61.9%)	6.50 (1.09–38.63)	0.04		

*dCMAP, compound muscle action potential with distal stimulation; pCMAP, compound muscle action potential with proximal stimulation; HFGS, Hughes Functional Grading Scale. Poor short-term outcome defined as inability to walk unaided (HFGS score ≥3) at discharge*.

**Table 4:** Univariate and multivariate regression analyses of factors of rapid recovery in the GBS patients after treatment.

**Demographic features**	**Number of patients**	**Number of patients with rapid recovery**	**Univariate OR (95% CI)**	***p*-value**	**Multivariate OR (95% CI)**	***p*-value**
Total	104	54 (51.9%)				
pCMAP				0.04		
0–0.40	47	19 (40.4%)	1			
0.40–1	32	17 (53.1%)	1.67 (0.68–4.13)			
>1	25	18 (72.0%)	3.79 (1.33–10.82)			
dCMAP				0.002		0.006
0–0.40	25	5 (20.0%)	1		1	
0.40–1	39	22 (56.4%)	5.18 (1.61–16.62)		5.10 (1.57–16.62)	
>1	40	27 (67.5%)	8.31 (2.55–27.10)		6.78 (2.02–22.72)	
AMAN	58	24 (41.4%)	0.38 (0.17–0.84)	0.017		

*dCMAP, compound muscle action potential with distal stimulation; pCMAP, compound muscle action potential with proximal stimulation. Rapid recovery defined as improvement of the HFGS score after 7 days of immunotherapy*.

**Table 5:** Univariate and multivariate regression analyses of factors of rapid recovery in AMAN subtype patients after treatment.

**Demographic features**	**Number of patients**	**Number of patients with rapid recovery**	**Univariate OR (95% CI)**	***p*-value**	**Multivariate OR (95% CI)**	***p*-value**
Total	58	24 (41.4%)				
dCMAP				0.02		0.02
0–0.40	18	3 (16.7%)	1		1	
0.40–1	25	11 (44.0%)	3.93 (0.90–17.10)		3.93 (0.90–17.08)	
>1	15	10 (66.7%)	10 (1.94–51.54)	0.006	10.00 (1.94–51.54)	0.01
pCMAP/dCMAP				0.094		
0.80–1.00	10	3 (30.0%)	1			
0.40–0.80	41	15 (36.6%)	1.35 (0.30–6.00)			
0–0.40	7	6 (85.7%)	14 (1.14–172.64)	0.04		

*dCMAP, compound muscle action potential with distal stimulation; pCMAP, compound muscle action potential with proximal stimulation. Rapid recovery defined as improvement of the HFGS score after 7 days of immunotherapy*.

## Discussion

The proportion and severity of the electrophysiological subtypes of GBS vary from region to region. Agreed with our previous study (1), this study revealed AMAN was still the most common subtype of GBS, occurring in 55.8% of patients, followed by AIDP (21.2%), as identified using the electrophysiological classification criteria of Rajabally et al. (8). Meanwhile, we found that most patients with antecedent diarrhea happened in AMAN subtype, while most patients with paresthesia and cranial nerve involvement happened in AIDP subtype, consistent with the reported characteristics of AMAN and AIDP (11–14). In our study, AMAN subtype had significantly longer hospitalization, the improvement of the HFGS score after 7 days of immunotherapy was slightly slower, but the severity of the disease and the mean dCMAP amplitude were almost similar in the AIDP and AMAN subtypes. In addition our results showed the decreased dCMAP is associated with slow recovery and poor outcome, which is consistent with the previous study (7) which further confirms that there is no difference in short-term prognosis and recovery between AIDP and AMAN subtypes in our region. These results agree with those of previous reports in our region (15). But in Europe, America, and Bangladesh, AMAN subtype showed a trend toward poorer recovery compared with AIDP (16); in southern China, a multicenter study showed that AMAN patients had higher HFGS score than AIDP patients (17); but in northeastern China, although AMAN had more severe symptoms than AIDP at admission, the prognosis was almost similar (12). This also suggests that not only the proportion of GBS subtype, but also the severity of the disease is different among regions, and even the pathological mechanism may be different.

On the other hand, we found that lower dCMAP, more days between onset and treatment, and higher HFGS score at admission were associated with poor short-term prognosis in all electrophysiological subtypes. For the AMAN subtype, antecedent diarrhea and a pCMAP/dCMAP amplitude <0.4 were strong factors for better short-term prognosis. The prognostic factors of the AMAN subtype are different. other studies identified antecedent diarrhea in elderly patients, lower Medical Research Council (MRC) sum score at admission and at day 7 after admission (2), low dCMAP amplitude (20% of the lower limit of the normal range) (7), and mechanical ventilation (18) as predictive of poor prognosis. Diarrhea was found to be a risk factor in some studies, mainly because diarrhea patients mostly present with AMAN, and these prognostic studies were concentrated in Europe and America, where the patients of AMAN subtype are more severe than AIDP and have poorer prognosis for the whole population including both AIDP and AMAN subtypes (2, 19). In our region, the AMAN subtype has similar clinical severity and prognosis as the AIDP (15, 20), no correlation between diarrhea and short-term prognosis was found in the analysis of the all population. Previous studies have shown that AMAN patients presented two different patterns of clinical recovery, rapid or slow (5, 21). Our results suggest that AMAN patients with antecedent diarrhea or CBs had better short-term prognosis. Although no previous study focused on the prognosis of AMAN subtypes, it was found in the population of northeastern China that absence of antecedent infections was associated with poor short-term prognosis in patients with mechanical ventilation (10). Meanwhile, a study in our region found that patients with lack of antecedent diarrhea were more likely to need mechanical ventilation support (22). These experimental results indirectly indicate that the patients with no antecedent infection (GBS triggered by non-infectious agents) have more severe clinical symptoms and worse prognosis, which is consistent with the more severe symptoms and poorer prognosis of GBS caused by gangliosides (non-infectious agents) in northeastern China and Spain (23, 24). Diarrhea is a factor for better short-term prognosis in AMAN patients mainly because these patients have very clear cause, after gastrointestinal infection, the body will produce antibodies against the axonal antigen, which may primarily attack peripheral nerve terminals lacking a blood-nerve barrier, causing local damage to the motor nerve terminals, and immunotherapy by IVIg can specifically neutralize the antibody. Furthermore, it was reported that, in our region, AMAN patients with CBs recover faster than those without CBs (25), which was also confirmed in our prognostic analysis. This may be because patients with CBs do not have significant axonal degeneration (26), and their axonal damage may be reversible.

In addition, we found 41 of 104 (39.4%) received both intravenous steroids and IVIg treatment, but there was no significant difference of HFGS score at nadir (*p* = 0.10) and discharge (*p* = 0.07) between the combination group and the IVIg group. There was also no statistical difference in the severity of HFGS score at admission (*p* = 0.48) and discharge (*p* = 0.43) in the use of the impact-dose steroid group compared to other unused or low-dose applications. Internationally, Both oral steroids and intravenous methylprednisolone are not recommended in GBS (27–29). Prior studies in Chinese centers showed that the combined use of intravenous corticosteroids with IVIg was not beneficial or potentially worse (30, 31). In our center, steroids may be used in early onset or severe cases (32), or where economic factors do not allow the use of IVIg. In the current study, our results once again suggest that the use of intravenous steroids has no benefit for patients with GBS.

Our experiments have several limitations. First, because of the single-center nature of the study, we have a limited number of patients and cannot include more factors; however, our sample size was sufficient for statistical analysis. Second, this study was a retrospective study in which information was extracted from medical records and long-term prognosis could not tracked. We only tracked short-term outcomes, and the prognostic factors were not validated based on long-term prognosis.

In conclusion, AMAN is still the most common type of GBS in our region, and is often triggered by diarrhea. Consistent with our electrophysiological results, AIDP and AMAN patients do not differ in their HFGS score at admission and discharge, but the recovery of the AMAN subtype is likely to be slow. Diarrhea and CBs are good outcome factors for the AMAN subtype.

## Data Availability

The datasets generated for this study are available on request to the corresponding author.

## Ethics Statement

This study was carried out in accordance with the recommendations of World Health Organization. WHO Guidelines on Ethical Issues in Public Health Surveillance, Research Ethics Committee of the Second Hospital of Hebei Medical University with written informed consent from all subjects. All subjects gave written informed consent in accordance with the Declaration of Helsinki. The protocol was approved by the Research Ethics Committee of the Second Hospital of Hebei Medical University.

## Author Contributions

JT and CC: conception and design of the study, analysis and interpretation of data, and drafting and revising of the manuscript. TL: clinical data collection and analysis. KZ: clinical data collection and manuscript revision. PL: clinical data analysis and statistical analysis. XL and YL: design and critical revision of the manuscript.

### Conflict of Interest Statement

The authors declare that the research was conducted in the absence of any commercial or financial relationships that could be construed as a potential conflict of interest.
